# Manifestation of subacute cutaneous lupus erythematosus during treatment with anti-PD-1 antibody cemiplimab – a case report

**DOI:** 10.3389/fimmu.2023.1324231

**Published:** 2023-12-08

**Authors:** Simon Fietz, Anne Fröhlich, Cornelia Mauch, Luka de Vos-Hillebrand, Tanja Fetter, Jennifer Landsberg, Friederike Hoffmann, Judith Sirokay

**Affiliations:** ^1^ Center for Skin Diseases Bonn, University Hospital Bonn, Bonn, Germany; ^2^ Center for Integrated Oncology, Cologne, Germany; ^3^ Center for Integrated Oncology, Bonn, Germany

**Keywords:** case report, cutaneous squamous cell carcinoma, immunotherapy, anti-PD-1 antibody, cutaneous lupus erythematosus

## Abstract

**Introduction:**

The anti-programmed cell death protein 1 (PD-1) antibody cemiplimab has shown promising results in the treatment of unresectable or metastatic squamous cell carcinoma, however, frequently leads to immune-related adverse events limiting therapy efficacy. Although cutaneous side effects are common, only very few cases of cutaneous lupus erythematosus have been reported under anti-PD-1 immunotherapy. So far, no case of cutaneous lupus has been described under treatment with cemiplimab.

**Case report:**

For the first time, we report the case of a patient with advanced squamous cell carcinoma, who developed clinical and histological findings in sun-exposed skin that were consistent with anti-SS-A/Ro antibody-positive subacute cutaneous lupus erythematosus (SCLE) under treatment with cemiplimab. Additionally, laboratory chemical analyses revealed a severe immune-related hepatitis without clinical symptoms. Both, the SCLE and the hepatitis, resolved after the administration of topical and systemic steroids and the discontinuation of anti-PD-1 therapy.

**Conclusion:**

Treatment with cemiplimab can be associated with the appearance of cutaneous lupus erythematosus in sun-exposed areas. Application of topical and systemic glucocorticoids can lead to a rapid resolution of the skin eruptions. Moreover, our case illustrates the possibility of simultaneously occurring severe immune-related adverse events. This highlights the importance of additional diagnostics to avoid overlooking additional immune-related adverse events.

## Introduction

**Figure 1:**
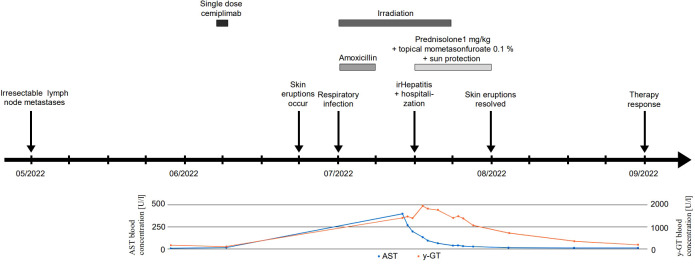
Timeline. Timeline of therapeutic regimens, patient’s symptoms, and laboratory studies.

The anti-programmed cell death protein 1 (PD-1) antibody cemiplimab has shown remarkable efficacy in the treatment of unresectable or metastatic cutaneous squamous cell carcinoma (CSCC) (1). However, immune-related adverse events frequently occur during anti-PD-1 treatment and can cause discontinuation of therapy (1). Subacute cutaneous lupus erythematosus (SCLE) is a rare but known adverse event during therapy with other anti-PD-1 antibodies, such as pembrolizumab or nivolumab (2). SCLE presents with erythematous, circular, scaly skin lesions on sun-exposed skin and is triggered by various drugs and UV light (3, 4). Diagnosis is based on skin lesion morphology, laboratory findings (e.g., anti-Ro (SS-A) antibodies) and histopathological findings (3, 4). Therapy includes sun protection and anti-inflammatory topical or systemic drugs, such as corticosteroids or hydroxychloroquine. Moreover, it should be considered to discontinue or pause the potentially triggering drug (3, 4). Cutaneous adverse events are very frequently in up to 50% of patients treated with anti-PD-1 therapy (1, 5–8). Among these, the appearance of SCLE was described for pembrolizumab and nivolumab in less than 0.01% of patients (9). In contrast to the vast majority of cutaneous adverse events, the appearance of SCLE frequently leads to discontinuation of therapy and thus constitutes a relevant potential adverse event (10–12). So far, no case of SCLE has been reported for treatment with cemiplimab (5). For the first time, we describe the occurrence of histopathologically and serologically confirmed SCLE in a patient undergoing treatment with cemiplimab (**Figure 1**).

## Case description

**Figure 2:**
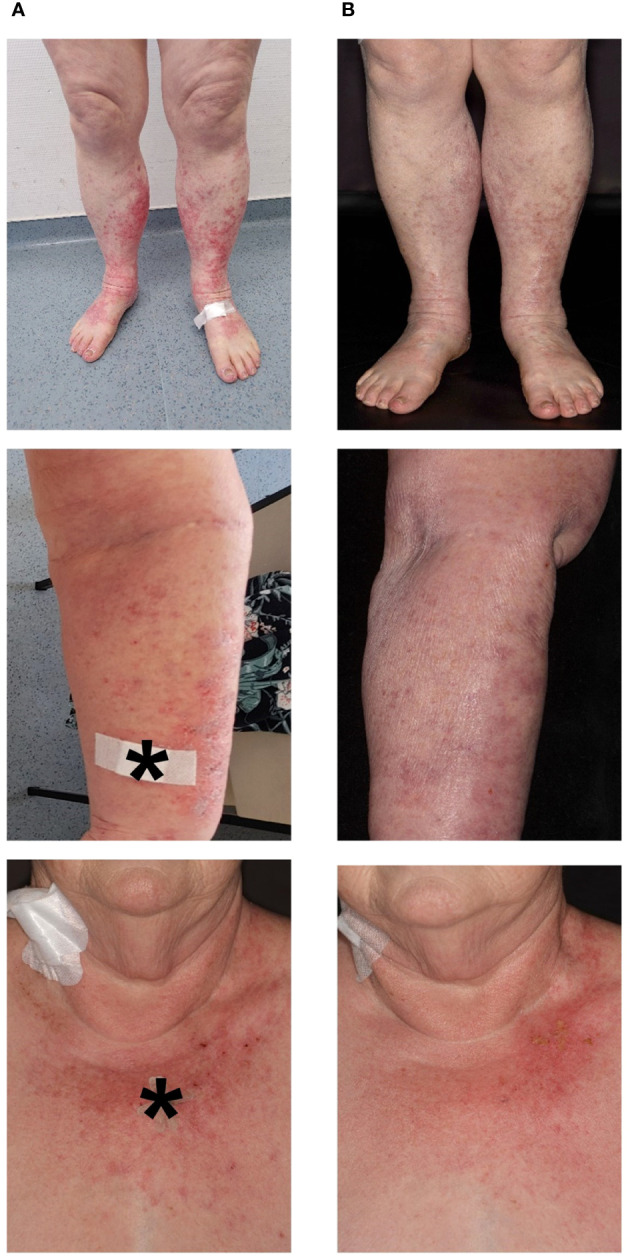
Clinical images **(A)** on initial contact and **(B)** 14 days after initiation of steroid treatment. Severely pruritic, scaly, coalescent, and erythematous macules and papules on sun-exposed skin (V-area of the upper trunk and neck, forearms, dorsal hands, lower legs, and dorsal feet). * indicates biopsy site.

A 64-year- old woman with lymphogenic metastasized CSCC of the left limb presented in our dermatological department with a three-week history of severely pruritic, coalescent, erythematous, and scaly macules and papules in sun-exposed areas of the skin (V-area of the upper trunk and neck, forearms, dorsal hands, lower legs, and dorsal feet, **Figure 2A**) five weeks after the administration of the anti-PD-1 antibody cemiplimab. The patient’s medical history included several benign and malignant light-induced skin eruptions (actinic keratoses, basal cell carcinoma) of the left limb, type 2 diabetes mellitus, and cardiovascular diseases, but no known autoimmune diseases. Despite the successful surgical removal of the primary tumor, the patient developed lymph node metastases of the left axilla (**Figure 1**). Because of disease progression that could not be managed surgically, we decided to initiate anti-PD-1 antibody therapy as first-line treatment in accordance with the current interdisciplinary guideline on invasive CSCC (13). Five weeks after the occurrence of lymph node metastases the patient received a single dose of cemiplimab 350 mg. Due to an infection of the upper airways three weeks after the first application, which resolved completely after treatment with Amoxicillin for seven days, cemiplimab treatment was halted. Additionally, three weeks after treatment initiation with cemiplimab, the patient underwent fractionated irradiation of symptomatic lymphogenic CSCC metastases of the left axilla for three weeks with a total dose of 40 Gray. The skin eruptions occurred two weeks after administration of cemiplimab and progressed over three weeks.

**Figure 3:**
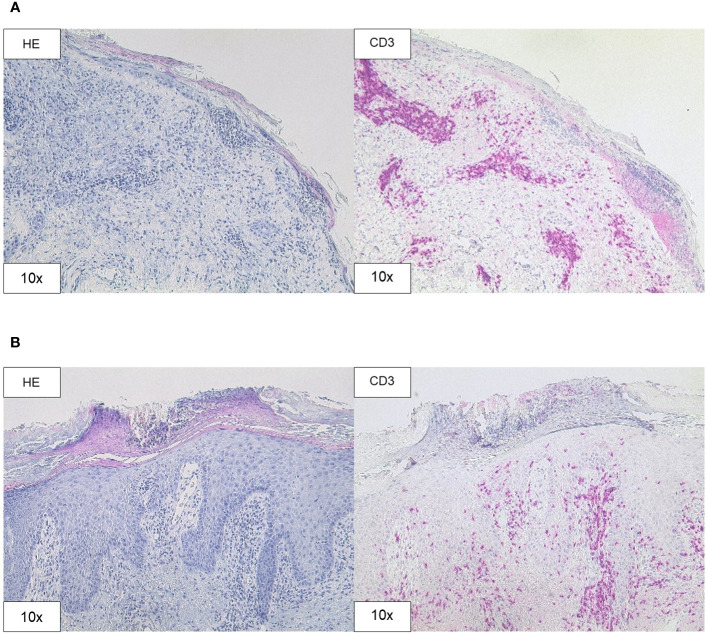
Histopathology. **(A)** H&E and CD3 staining (original magnification 10x) of the skin biopsy of the chest demonstrating interface dermatitis with colloid bodies, perifollicular mucin, and mixed immune cells, containing lymphocytes, histiocytes, and neutrophil granulocytes. **(B)** H&E and CD3 staining (original magnification 10x) of the skin biopsy of the right forearm showing acanthosis, parakeratosis, intraepithelial neutrophil granulocytes, and CD3 positive T cell infiltration.

A skin biopsy of the chest showed an interface dermatitis with colloid bodies, perifollicular mucin, and mixed immune cells (**Figure 3A**). A second skin biopsy of the right forearm displayed an acanthosis, parakeratosis, and intraepithelial neutrophil granulocytes, consistent with a psoriasiform inflammation (**Figure 3B**). Immunoblotting revealed highly positive anti-Ro-52 (SS-A) antibodies (299 U/ml using ELISA), whereas direct immunofluorescence did not show precipitations of IgA, IgG, IgM, C3, or fibrin. Routine blood testing revealed highly elevated liver enzymes and cholestasis parameters (peak values: AST 401 U/l, ALT 267 U/l; γ-GT 1947 U/l, alkaline phosphatase 640 U/l, total bilirubin 2.56 mg/dl; **Figure 1**) without any pathologies in the clinical examination and computed tomography imaging of the liver. Hepatitis virus serology as well as autoimmune hepatitis panel were negative. The patient was diagnosed with drug-induced, immune-related SCLE, Common Terminology Criteria for Adverse Events version 5.0 (CTCAE v5.0) grade 2 and immune-related hepatitis, CTCAE v5.0 grade 3 (14). Treatment with prednisolone for the severe hepatitis and topical steroids (mometasonfuroate 0.1% ointment) were initiated and tapered over eight weeks. The cutaneous eruptions gradually resolved (**Figure 2B**) as well as the elevated liver enzymes and cholestasis parameters (**Figure 1**). Due to the severe CTCAE v5.0 grade 3 hepatitis we decided not to re-initiate treatment with cemiplimab according to the current ASCO guideline (15). Imaging after three months showed therapy response and the patient remained progression-free for eight months.

## Discussion

For the first time, we present the rare occurrence of SCLE related to cemiplimab therapy complicated by immune-related hepatitis. As described by Marzano et al. drug-induced SCLE usually goes along with elevated anti-Ro (SS-A)-antibodies and is chronologically related to the triggering drug (3). Because of the typical clinical appearance and distribution of the skin eruptions and the high concentration of anti-Ro (SS-A)-antibodies, we diagnosed SCLE despite the lack of lesional immune precipitates. The skin biopsies taken from the chest and the right forearm revealed an interface dermatitis and a psoriasiform inflammation, respectively. Marzano et al. described two different predominant phenotypic variants of drug-induced SCLE: ‘papulosquamous’ and ‘annular polycyclic’ (3). SCLE mostly goes along with histological interface dermatitis (3). However, biopsies taken from ‘papulosquamous’ SCLE can show psoriasiform changes as well (4, 5). In accordance with the clinical phenotype both biopsies contribute to a ‘papulosquamous’ SCLE in the case of our patient.

It remains unclear whether a topical application of glucocorticoids would have been sufficient for the recovery of the skin lesions, as systemic steroid treatment was necessary to treat the concomitant immune-related hepatitis. In patients treated with the anti-PD-1 antibodies pembrolizumab or nivolumab moderate SCLE eruptions could be treated successfully by discontinuation of immunotherapy and were kept under control after re-initiation of systemic treatment by the use of topical steroids (16, 17). In addition to treatment discontinuation and topical steroids the use of systemic hydroxychloroquine and/or glucocorticoids alone or in combination was described to resolve severe SCLE skin eruptions (10–12, 18–23). By this means, immunotherapy could be continued successfully in some cases without or with only a mild relapse of SCLE (20–22). Although the patient only underwent irradiation of the left axilla, radiotherapy is a known trigger of cutaneous lupus erythematosus and was temporally related to the occurrence of the presented skin eruptions in our case (24, 25). Therefore, irradiation could have acted as a trigger for SCLE in the described case. Moreover, the simultaneous irradiation of the metastatic site could have masked response to cemiplimab. In our case, cemiplimab treatment was discontinued because of the severe hepatitis. Monitoring of SCLE and evaluation of possible therapy options under cemiplimab treatment might be of interest for future patients.

In conclusion, our case demonstrates that patients receiving cemiplimab can develop cutaneous lupus erythematosus. The concomitant immune-related hepatitis underlines the need to screen for additional adverse events other than skin conditions. Diagnosis and treatment of patients with adverse events on cemiplimab treatment will be a challenge for dermatologists in the future.

## Patient perspective

For the patient, an improvement of the sever pruritus accompanying the cutaneous lesions was of primary importance. As she did not develop any clinical symptoms from the hepatitis, it was challenging for the patient to stay motivated to continue on systemic steroids after resolution of the skin symptoms. Transparent communication of diagnostic results and therapeutic consequences was important for the patient to reach a high level of compliance.

## Data availability statement

The data analyzed in this study is subject to the following licenses/restrictions: a publication of datasets containing original patient-related data could affect the patient’s anonymization. Original datasets are available from the corresponding author on reasonable request. Requests to access these datasets should be directed to Simon Fietz, Simon.Fietz@ukbonn.de.

## Ethics statement

Written informed consent was obtained from the individual(s) for the publication of any potentially identifiable images or data included in this article.

## Author contributions

SF: Conceptualization, Formal Analysis, Investigation, Visualization, Writing – original draft, Writing – review & editing, Data curation, Project administration, Software, Validation. AF: Supervision, Writing – original draft. CM: Conceptualization, Supervision, Writing – original draft. LV: Supervision, Writing – original draft. TF: Data curation, Investigation, Writing – original draft. JL: Supervision, Writing – original draft. FH: Conceptualization, Project administration, Supervision, Writing – original draft. JS: Conceptualization, Project administration, Supervision, Writing – original draft.
